# Clinical outcomes of angiosarcoma: a single institution experience

**DOI:** 10.1186/s40880-019-0389-1

**Published:** 2019-08-06

**Authors:** Jae-Joon Kim, Seyoung Seo, Jeong Eun Kim, Sung-Ho Jung, Si Yeol Song, Jin-Hee Ahn

**Affiliations:** 1 Department of Oncology, Asan Medical Center, University of Ulsan College of Medicine, 88, Olympic-ro 43-gil, Songpa-gu, Seoul, 05505 South Korea; 2 Division of Hematology and Oncology, Pusan National University Yangsan Hospital, Yangsan, 50612 South Korea; 3 Department of Thoracic and Cardiovascular Surgery, Asan Medical Center, University of Ulsan College of Medicine, Seoul, 05505 South Korea; 4 Department of Radiation Oncology, Asan Medical Center, University of Ulsan College of Medicine, Seoul, 05505 South Korea

Dear Editor,

Angiosarcoma is a rare malignancy, which accounts for approximately 1%–2% of soft-tissue sarcomas [1]. It can occur at any site within the human body. Some researchers suggested that site distribution may be different between Eastern Asian and Western countries [1]. Several factors, such as age, stage at diagnosis, performance status, site of the disease, histological grade, tumor size, and visceral involvement, were suggested as prognostic factors in angiosarcoma [2–5]. However, there is still controversy regarding to which prognostic factors are significantly important. As there are no randomized clinical trials for angiosarcoma, its treatments are guided based on guidelines of soft-tissue sarcoma or the results of several retrospective studies. Radical surgery is the preferred treatment for angiosarcoma [2–4]. Some studies suggested that receiving adjuvant chemotherapy (CT) is associated with better outcome [5], while other studies have shown that adjuvant CT has no significant effect [6, 7]. Chemoradiotherapy has been reported to be effective in angiosarcoma especially for cutaneous angiosarcoma [8]. However, evidence for the efficacy and safety of palliative cytotoxic CT is still limited. Therefore, here, we present our analysis based on the clinical features, tumor characteristics, treatment and outcomes of angiosarcoma patients in an attempt to investigate the significant prognostic factors and efficient therapeutics in real clinical practice.

A total of 89 patients who were diagnosed with angiosarcoma at the Asan Medical Center (Seoul, Korea) between January 1992 and December 2014were evaluated. The median age at diagnosis was 60 years (range 20–85 years), and 69.7% (*n* = 62) of the patients were men. Forty-three (48.3%) patients had localized tumor at the time of diagnosis, whereas 46 (51.7%) patients had advanced disease (7 patients with locally advanced angiosarcoma, 39 patients with metastatic disease). The locations of the tumors at diagnosis were variable. The head and neck location including the scalp (24 cases, 27.0%) was the most common primary site. The tumor location of 22 (24.7%) and 15 (16.9%) patients were at the liver and heart, respectively. Twelve (13.5%) cases were at the trunk including three cases each located at the lung, pulmonary artery, and ovary, and one case each at the chest wall, diaphragm, and pleura. There were 7 cases (7.9%) originated from the spleen and 4 (4.5%) breast angiosarcoma. The number of cases of angiosarcoma located at the extremities, bone, and nasal cavity were 2 (2.2%), 2 (2.2%) and 1 (1.1%), respectively (Additional file 1: Table S1).

Thirty-nine (43.8%) patients were treated with curative intent treatments, including 20 patients with surgery only, 9 patients with surgery and radiotherapy (RT), 7 patients with surgery and CT, and 3 patients with surgery, RT and CT. Thirty-five (39.3%) patients were treated in palliative setting, including 20 patients with palliative CT only (1 patient with localized tumor and 19 patients with advanced disease), 8 patients with palliative surgery only (1 patient with localized tumor and 7 patients with advanced disease), 4 patients with surgery and CT, 2 patients with RT only (1 patient with localized tumor and 1 patient with advanced disease), and 1 patients with CT and RT. Twelve (13.5%) advanced patients were treated with only best supportive care (1 patient with localized tumor and 11 patients with advanced disease) (Additional file 2: Table S2).

The observed progression-free survival (PFS) and overall survival (OS) were different for different primary tumor site. The median PFS was longest in angiosarcoma located at the trunk (9.0 months; range 0.9–37.3 months; 95% confidence interval [CI] 0.0–18.5), followed by the breast (7.6 months; range 4.8–82.0 months; 95% CI 3.8–11.4), heart (4.8 months; range 0.1–30.8 months; 95% CI 3.2–6.4), scalp or head and neck (4.1 months; range 1.4–95.4 months; 95% CI 3.0–5.2), spleen (3.3 months; range 0.7–87.6 months; 95% CI 0.0–8.1), liver (2.3 months; range 0.1–40.1 months; 95% CI 1.8–2.8), and others. The median OS was longest in patients with angiosarcoma located at the trunk (21.7 months; range 0.9–67.0 months; 95% CI 7.2–36.2), followed by the breast (21.6 months; range 8.8–88.9 months; 95% CI not available [NA]), scalp or head and neck (14.2 months; range 1.8–105.3 months; 95% CI 5.1–23.2), heart (11.3 months; range 0.1–38.3 months; 95% CI 8.7–14.0), spleen (3.8 months; range 0.9–94.6 months; 95% CI 0.3–7.2), liver (2.6 months; range 0.1–46.5 months; 95% CI 0.0–5.4), and others. With median follow-up of 10.3 months (range 0.1–179.3 months) in surviving patients, the 1-year OS rate was 43.4%. In univariate analysis, poor performance status, advanced disease, and primary liver angiosarcoma were associated with shorter PFS (*P* < 0.001, *P* < 0.001 and *P* = 0.035, respectively). In regard to OS, age above 60 years, poor performance status, advanced disease and primary liver angiosarcoma were associated with poor survival (*P* = 0.011, *P* < 0.001, *P *< 0.001 and *P* = 0.017, respectively). Multivariate analyses showed that poor performance and advanced disease were significant prognostic factors for PFS (*P* = 0.002 and *P* = 0.007, respectively), while old age, poor performance, and advanced disease were significant prognostic factors for OS (*P *< 0.001, *P* = 0.003 and *P* < 0.001, respectively) (Additional file 3: Table S3).

For the 43 patients with localized angiosarcoma, their median PFS and OS was 8.6 months (95% CI 3.4–13.9) and 21.6 months (95% CI 12.2–30.1), respectively. Among them, 39 (90.7%) patients had curative surgery. Four (9.3%) patients did not undergo surgery because of the patient’s refusal (*n* = 3) or difficulty for surgical resection (*n* = 1). In patients with localized tumors, those who had curative resection demonstrated significantly better PFS (8.7 months; [95% CI 3.3–14.1] vs. 3.3 months [95% CI NA], *P *= 0.050) and OS (23.7 months [95% CI 15.5–31.8] vs. 8.8 months, [95% CI 6.8–10.7], *P *< 0.001) than those who did not had curative operation. The median PFS and OS of patients who had curative surgery only were 6.9 months (95% CI 2.6–11.3) and 21.6 months (95% CI 5.4–37.8), respectively. The median PFS of patients who had curative surgery and CT were 30.8 months (range 13.2–48.5 months), which was numerically the longest compared to the patients who received other treatment. In these patients, the primary tumor sites were the ovary (*n* = 3), right atrium of heart (*n* = 2), lung (*n* = 1), and pulmonary artery (*n* = 1). For the 46 patients with advanced disease, their median PFS and OS were 3.4 months (range 2.5–4.3 months) and 4.7 months (range 3.4–6.4 months), respectively (Additional file 2: Table S2).

A total of 20 patients received palliative CT (9 received doxorubicin-based regimens, 9 received paclitaxel-based regimens, and 2 received cisplatin-based regimens). Regardless of the regimens prescribed, patients who received palliative CT had significantly longer PFS (*P *= 0.030) and OS (*P *= 0.001) than those who were treated with best supportive care (Additional file 4: Fig. S1). However, there were no significant differences in PFS (*P* = 0.179) and OS (*P* = 0.221) between the patients who received different first-line palliative CT regimens.

In the present study, scalp or head and neck angiosarcomas were the most common locations for angiosarcomas, which was consistent with previous reports [1]. The next frequent primary sites, mentioned in an orderly sequence from common to least common, were the liver, heart, and trunk. The frequency of angiosarcoma locations has been reported slightly differently in previous literature. The frequency of primary tumor locations in this study was similar to that of the studies from Asian countries [1, 9] but different to that of Western countries, where there are less frequent cases of liver (range 1.8%–6.0%) angiosarcomas but more frequently extremities-located angiosarcomas (range 15.3%–52.0%) [2, 3].

The prognosis of angiosarcoma is worse than that of primary soft tissue sarcomas. The 5-year survival rate of angiosarcomas is about 40% [3]. As the definitive prognostic factors for angiosarcomas are yet unclear, we performed this study to clarify this dilemma. We found that older age, poor performance status and advanced disease were associated with shorter PFS and OS. Comparatively, several prior studies have suggested similar findings in which old age, metastatic disease at presentation, poor performance status and site of the disease could also affect prognosis [3–5]. Therefore, our data is in concordance with these existing evidences. In contrast, there have been some studies which have suggested that tumor grade might also be associated with the prognosis [2], however, this was not conclusive as there have been studies presented with conflicting results [4].

In the present study, even though the median PFS and OS of patients who were treated with curative surgery and adjuvant CT were numerically the longest, the efficacy of adjuvant CT was still questionable due to potential bias from the small sample size and heterogenous primary tumor locations. In a palliative setting, Chen et al. [9] demonstrated that patients who received palliative CT demonstrated longer OS than those who did not receive it. Similarly, in our study, we have found that patients who received palliative CT had better prognosis, including PFS and OS than those with best supportive care. We believe that we need to consider palliative CT for angiosarcoma patients based on this study even though it was retrospective data.

This study has several limitations. Due to some particular characteristics of angiosarcoma including its rarity, diverse primary sites and variety of treatment strategies, it was hard to analyze the impact of each factor. Furthermore, because clinical data were collected during a long period, retrospectively, the treatment strategies were diverse and not unified, which made comparing effective treatment strategies difficult. However, as there still have been small numbers of literature concerning the prognostic factors and treatment of angiosarcoma, the findings from this study can be helpful for predicting the outcome and determining therapeutic options for angiosarcoma patients.

Recently, novel therapies in patients with angiosarcoma have been reported. Immunotherapies including immune checkpoint inhibitors demonstrated promising response in patients with cutaneous angiosarcoma. Targeted therapy which inhibits endoglin showed promising antitumor activities in combination with pazopanib in early phase clinical trials [10]. A number of clinical trials with novel agents are currently ongoing. As palliative CT can be helpful to patients with angiosarcoma, it would be important to develop new therapeutic strategies with various novel drugs through clinical trials.

In conclusion, the present study showed that the distribution of angiosarcoma in Eastern countries was different from those of Western countries. Older age, poor performance status, and advanced disease were poor prognostic predictors. For the patients with unresectable or metastatic disease, palliative CT could be beneficial with better survival outcome as compared to best supportive care.

## Additional files

**Additional file 1: Table S1.** Characteristics of 89 patients with angiosarcoma.

**Additional file 2: Table S2.** Treatment of 89 patients with primary angiosarcoma.

**Additional file 3: Table S3.** Univariate and multivariate analysis of patients with angiosarcoma for PFS and OS.

**Additional file 4: Fig. S1.** Survival curves according to whether the patients received palliative chemotherapy or best supportive care. Kaplan–Meier curve of progression-free survival (A) and overall survival (B).

## Data Availability

The datasets used in the present study are available from the corresponding author on reasonable request.

## Abbreviations

RT: radiotherapy
CT: chemotherapy
PFS: progression-free survival
OS: overall survival
CI: confidence interval
ECOG PS: Eastern Cooperative Oncology Group performance status
HR: hazard ratio

## References

[1] Naka N, Ohsawa M, Tomita Y, Kanno H, Uchida A, Aozasa K (1995). Angiosarcoma in Japan. a review of 99 cases. Cancer.

[2] Abraham JA, Hornicek FJ, Kaufman AM, Harmon DC, Springfield DS, Raskin KA (2007). Treatment and outcome of 82 patients with angiosarcoma. Ann Surg Oncol.

[3] Fayette J, Martin E, Piperno-Neumann S, Le Cesne A, Robert C, Bonvalot S (2007). Angiosarcomas, a heterogeneous group of sarcomas with specific behavior depending on primary site: a retrospective study of 161 cases. Ann Oncol.

[4] Pawlik TM, Paulino AF, McGinn CJ, Baker LH, Cohen DS, Morris JS (2003). Cutaneous angiosarcoma of the scalp: a multidisciplinary approach. Cancer.

[5] Naka N, Ohsawa M, Tomita Y, Kanno H, Uchida A, Myoui A (1996). Prognostic factors in angiosarcoma: a multivariate analysis of 55 cases. J Surg Oncol.

[6] Woll PJ, Reichardt P, Le Cesne A, Bonvalot S, Azzarelli A, Hoekstra HJ (2012). Adjuvant chemotherapy with doxorubicin, ifosfamide, and lenograstim for resected soft-tissue sarcoma (EORTC 62931): a multicentre randomised controlled trial. Lancet Oncol..

[7] Sarcoma Meta-analysis Collaboration (1997). Adjuvant chemotherapy for localised resectable soft-tissue sarcoma of adults: meta-analysis of individual data. Lancet.

[8] Fujisawa Y, Yoshino K, Kadono T, Miyagawa T, Nakamura Y, Fujimoto M (2014). Chemoradiotherapy with taxane is superior to conventional surgery and radiotherapy in the management of cutaneous angiosarcoma: a multicentre, retrospective study. Br J Dermatol.

[9] Chen TWW, Pang A, Puhaindran ME, Maw MM, Loong HH, Sriuranpong V (2016). Optimal first line systemic therapy in patients (pts) with metastatic angiosarcoma: a report from the Asian Sarcoma Consortium [ESMO abstract 502O_PR]. Ann Oncol.

[10] Florou V, Wilky BA (2018). Current and future directions for angiosarcoma therapy. Curr Treat Options Oncol.

