# Supplementary material for: Clinical outcomes of angiosarcoma: a single institution experience
Source: Cancer Commun (Lond). 2019 Aug 6;39:44. doi: 10.1186/s40880-019-0389-1 (PMC6685159; doi:10.1186/s40880-019-0389-1)
Supplement: Supplementary file 1 — Additional file 1: Table S1. Characteristics of 89 patients with angiosarcoma. [file 40880_2019_389_MOESM1_ESM.docx]

**Additional file 1: Table S1.** Characteristics of 89 patients with angiosarcoma.

| Characteristic | No. of cases (%) |
| --- | --- |
| Age [years; median (range)] | 60 (20-85) |
|  |  |
| ≤60 | 42 (47.2) |
| >60 | 47 (52.8) |
| Sex |  |
| Male | 62 (69.7) |
| Female | 27 (30.3) |
| ECOG PS score | |
| 0 | 6 (6.7) |
| 1 | 66 (74.2) |
| 2 | 13 (14.6) |
| 3 | 4 (4.5) |
| 4 | 0 |
| Extent of disease |  |
| Localized tumor | 43 (48.3) |
| Advanced disease | 46 (51.7) |
| History of previous radiation  Present  Absent | 2 (2.2)  87 (97.8) |
| History of lymphedema  Present  Absent | 3 (3.4)  86 (96.6) |
| Living status |  |
| Alive without disease | 7 (7.9) |
| Alive with disease | 3 (3.4) |
| Dead | 79 (88.8) |
| Primary tumor location |  |
| Scalp, Head and neck | 24 (27.0) |
| Liver | 22 (24.7) |
| Heart | 15 (16.9) |
| Trunk | 12 (13.5) |
| Spleen | 7 (7.9) |
| Breast | 4 (4.5) |
| Extremities | 2 (2.2) |
| Bone | 2 (2.2) |
| Nasal cavity | 1 (1.1) |

ECOG PS, Eastern Cooperative Oncology Group performance status.
