# Supplementary material for: Clinical outcomes of angiosarcoma: a single institution experience
Source: Cancer Commun (Lond). 2019 Aug 6;39:44. doi: 10.1186/s40880-019-0389-1 (PMC6685159; doi:10.1186/s40880-019-0389-1)
Supplement: Supplementary file 2 — Additional file 2: Table S2. Treatment of 89 patients with primary angiosarcoma. [file 40880_2019_389_MOESM2_ESM.docx]

**Additional file 2: Table S2.** Treatment of 89 patients with primary angiosarcoma.

| Type of treatment | Number of cases  (%) | PFS  [months, median (95% CI)] | OS  [months, median (95% CI)] |
| --- | --- | --- | --- |
| Localized tumor | 43 (100) | 8.6 (3.4-13.9) | 21.6 (12.2-30.1) |
| Curative surgery | 20 (46.5) | 6.9 (2.6-11.3) | 21.6 (5.4-37.8) |
| Curative surgery + RT | 9 (20.9) | 4.1 (3.7-4.4) | 14.6 (14.2-15.0) |
| Curative surgery + CT^a^ | 7 (16.3) | 30.8 (13.2-48.5) | 24.0 (4.0-44.0) |
| Curative surgery + CT^b^ + RT | 3 (7.0) | 8.7 (6.9-10.5) | 49.2 (NA) |
| Palliative RT | 1 (2.3) | 3.7 (NA) | 7.1 (NA) |
| Palliative CT^c^ | 1 (2.3) | NA | 11.4 (NA) |
| Palliative surgery | 1 (2.3) | 3.3 (NA) | 9.1 (NA) |
| Best supportive care | 1 (2.3) | NA | 8.8 (NA) |
| Advanced disease | 46 (100) | 3.4 (2.5-4.3) | 4.7 (3.4-6.4) |
| Palliative CT^d^ | 19 (41.3) | 3.9 (2.7-5.0) | 7.7 (1.6-13.7) |
| Palliative surgery | 7 (15.2) | 1.8 (0.0-4.6) | 1.8 (0.0-4.1) |
| Palliative surgery + CT^e^ | 4 (8.7) | 3.4 (2.7-4.1) | 5.5 (0.0-11.6) |
| Palliative CT^f^ + RT | 1 (2.2) | 7.3 (NA) | 11.3 (NA) |
| Palliative RT | 1 (2.2) | 3.1 (NA) | 3.1 (NA) |
| Best supportive care | 11 (23.9) | 2.2 (0.1-4.3) | 2.2 (0.3-4.1) |
| Unknown | 3 (6.5) | 2.3 (0.3-4.3) | 4.7 (0.4-9.0) |

CT, chemotherapy; RT, radiotherapy; PFS, progression-free survival; OS, overall survival; CI, confidence interval, NA, not available.

^a^ 2 patients had doxorubicin-based CT, 2 had paclitaxel-based CT, 3 had cisplatin-based CT, 1 had ifosfamide-based CT;

^b^ 3 patients had oxorubicin-based CT;

^c^ 1 patient had paclitaxel-based CT;

^d^ 9 patients had doxorubicin-based CT, 8 had paclitaxel-based CT, 2 had cisplatin-based CT;

^e^ 3 patients had doxorubicin-based CT, 1 had cisplatin-based CT,

^f^1 patient had cisplatin-based CT.
