# Supplementary material for: Clinical outcomes of angiosarcoma: a single institution experience
Source: Cancer Commun (Lond). 2019 Aug 6;39:44. doi: 10.1186/s40880-019-0389-1 (PMC6685159; doi:10.1186/s40880-019-0389-1)
Supplement: Supplementary file 3 — Additional file 3: Table S3. Univariate and multivariate analysis of patients with angiosarcoma for PFS and OS. [file 40880_2019_389_MOESM3_ESM.docx]

**Additional file 3: Table S3.** Univariate and multivariate analysis of patients with angiosarcoma for PFS and OS.

|  | PFS | | | | OS | | | | |
| --- | --- | --- | --- | --- | --- | --- | --- | --- | --- |
|  | Univariate analysis | | Multivariate analysis | | Univariate analysis | | Multivariate analysis | | |
|  | HR (95% CI) | *P* value | HR (95% CI) | *P* value | HR (95% CI) | *P* value | | HR (95% CI) | *P* value |
| Age (years; ≤60 *vs.* >60) | 1.590  (0.999-2.528) | 0.050 |  |  | 1.786  (1.139-2.799) | 0.011 | | 2.720  (1.631-4.535) | <0.001 |
| Sex (male *vs.* female) | 0.778  (0.471-1.286) | 0.327 |  |  | 0.651  (0.393-1.078) | 0.095 | |  |  |
| ECOG PS (0-1 *vs.* 2-4) | 3.830  (2.134-6.874) | <0.001 | 2.539  (1.405-4.588) | 0.002 | 4.192  (2.383-7.377) | <0.001 | | 2.600  (1.387-4.875) | 0.003 |
| Extent of disease (localized tumor *vs.* advanced disease) | 2.651  (1.639-4.287) | <0.001 | 1.976  (1.201-3.250) | 0.007 | 3.270  (2.048-5.223) | <0.001 | | 3.632  (2.038-6.471) | <0.001 |
| Visceral organ origin  (no *vs.* yes) | 1.346  (0.829-2.185) | 0.230 |  |  | 1.564  (0.974-2.511) | 0.064 | |  |  |
| Scalp, Head and neck origin (no *vs.* yes) | 0.847  (0.532-1.486) | 0.525 |  |  | 0.843  (0.517-1.372) | 0.491 | |  |  |
| Liver origin (no vs. yes) | 1.727  (1.040-2.869) | 0.035 | 1.230  (0.737-2.053) | 0.429 | 1.973  (1.200-3.244) | 0.017 | | 1.281  (0.755-2.174) | 0.358 |
| Heart origin (no vs. yes) | 1.302  (0.723-2.342) | 0.379 |  |  | 1.323  (0.766-2.411) | 0.338 | |  |  |
| Trunk origin (no vs. yes) | 1.626  (0.806-3.278) | 0.174 |  |  | 0.593  (0.307-1.235) | 0.141 | |  |  |
| Spleen origin (no vs. yes) | 0.932  (0.375-2.315) | 0.879 |  |  | 0.995  (0.449-2.386) | 0.991 | |  |  |
| Breast origin (no vs. yes) | 0.540  (0.170-1.720) | 0.297 |  |  | 0.180  (0.026-1.348) | 0.089 | |  |  |

ECOG PS, Eastern Cooperative Oncology Group performance status; HR hazard ratio, CI, confidence interval; OS, overall survival; PFS, progression-free survival
