# Supplementary material for: Clinical outcomes of angiosarcoma: a single institution experience
Source: Cancer Commun (Lond). 2019 Aug 6;39:44. doi: 10.1186/s40880-019-0389-1 (PMC6685159; doi:10.1186/s40880-019-0389-1)
Supplement: Supplementary file 4 — Additional file 4: Fig. S1. Survival curves according to whether the patients received palliative chemotherapy or best supportive care. Kaplan–Meier curve of progression-free survival (A) and overall survival (B). [file 40880_2019_389_MOESM4_ESM.docx]

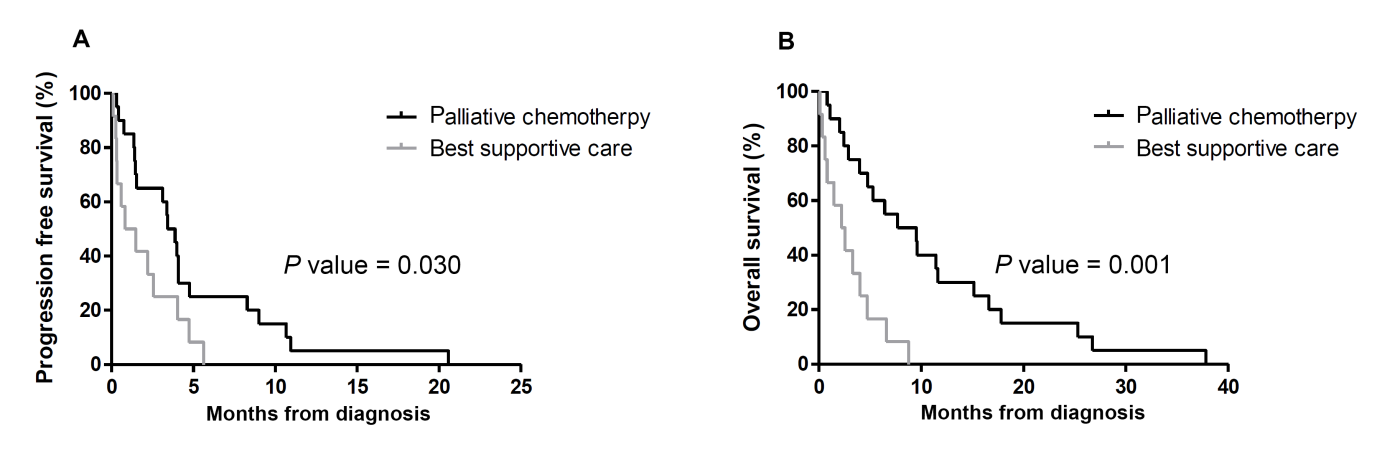


Additional file 4: Fig. S1. Survival curves according to whether the patients received palliative chemotherapy or best supportive care. Kaplan-Meier curve of progression-free survival (A) and overall survival (B).
